# Speed of Sound Measurements in Helium at Pressures from 15 to 100 MPa and Temperatures from 273 to 373 K

**DOI:** 10.1021/acs.jced.3c00083

**Published:** 2023-05-05

**Authors:** Carsten Wedler, J. P. Martin Trusler

**Affiliations:** Department of Chemical Engineering, Imperial College London, London SW7 2AZ, U.K.

## Abstract

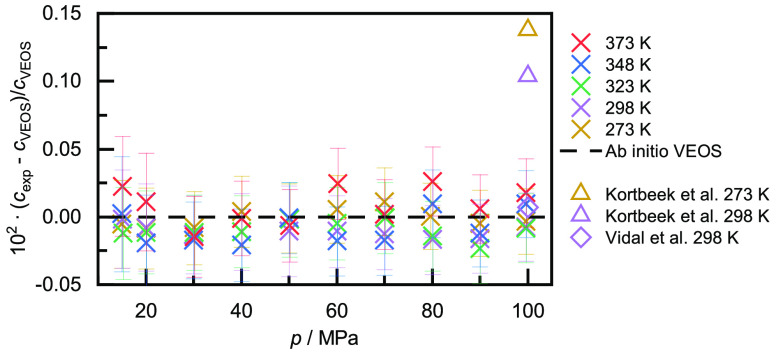

The speed of sound in helium was measured along five isotherms in a temperature range from 273 to 373 K at pressures from 15 to 100 MPa with a relative expanded uncertainty (*k* = 2) from 0.02 to 0.04%. A dual-path pulse-echo system was utilized to conduct these measurements. The data were compared with the reference equation of state developed by Ortiz Vega et al. At pressures up to 50 MPa, relative deviations were within the uncertainty of our measurements, while, at higher pressures, increasing negative deviations were observed up to -0.26%. We also compared the results with predictions based on the virial equation of state correct to the seventh virial coefficient, using the *ab initio* virial coefficients reported recently by Gokul et al., finding agreement to within the experimental uncertainty at all investigated states.

## 1 Introduction

Helium is a noble gas with numerous important applications in science and industry. Liquid helium is a crucial cryogen for low-temperature applications, especially cooling of superconducting magnets.^1,2^ Due to its non-toxic nature and properties that differ markedly from air, it is a perfect gas for leak detection in high-pressure systems.^3^ In adsorption science, helium is extensively used as an inert gas to determine the skeletal volume of adsorbent materials.^4−6^ It is also used as a carrier gas in gas chromatography.^7^ As it is the only gas with small dimensions, low density, high speed of sound, and low boiling point similar to hydrogen, it is also a perfect gas for calibrating instruments used in connection with hydrogen storage, transport, and utilization. Regarding hydrogen liquefaction, helium is an important refrigerant, both in pure form and as part of a mixture (e.g., helium/neon mixtures).^8,9^ For all these applications, a reliable description of the thermophysical properties of helium is necessary. Therefore, an equation of state (EOS) to calculate thermodynamic properties of ^4^He was developed in 1990 by McCarty and Arp.^10^ In 2013, Ortiz Vega^11^ published in his PhD thesis a fundamental Helmholtz energy EOS which describes the thermodynamic properties of helium more accurately than the EOS of McCarty and Arp.^10^ However, in common thermophysical property software such as REFPROP 10.0^12^ or TREND 5.0,^13^ the EOS from the PhD thesis by Ortiz Vega^11^ is superseded by an improved but unpublished EOS, also due to Ortiz Vega et al.^14^ This EOS is dated 2015 and is called the “final” EOS in both software and is also the EOS recommended for helium by NIST.^12^ In the PhD thesis of Ortiz Vega,^11^ the only speed of sound data considered for gaseous helium were those of Gammon^15^ and Hurly et al.,^16^ which cover pressures below about 15 MPa. Thus, the EOS was not validated for pressures above 15 MPa. The author gives an uncertainty for the calculation of the speed of sound of about 0.02%, as the EOS describes the data of Gammon^15^ with deviations within ±0.02%. For the unpublished ’final’ EOS by Ortiz Vega et al.,^14^ REFPROP 10 and TREND 5.0 specify that the relative uncertainty of the speed of sound in the vapor phase is 0.01%. It is not obvious if the “final” EOS was optimized against the same data set or if additional speed of sound data was considered. However, to the best of our knowledge, no new speed of sound data in gaseous helium were published after the initial publication of Ortiz Vega.^11^

A review of speed of sound data in gaseous helium available in the literature is presented in Table 1. Absolute average relative deviations (AARD) of the experimental data from values calculated using REFPROP 10 with the unpublished EOS of Ortiz Vega et al.^14^ are given. The data sets of Gammon^15^ and Hurly et al.,^16^ which were used for the fitting procedure described in the PhD thesis of Ortiz Vega,^11^ show by far the lowest AARD. Therefore, it seems likely that both data sets were used to adjust the unpublished EOS of Ortiz Vega et al.^14^ The low- and ultra-low-pressure data reported by Van Itterbeek and co-workers^17,18^ show AARDs below 0.1%. However, all other reported data show significant deviations. In addition, it becomes apparent that there is a gap in the literature for pressures of 15 to 100 MPa, as there are only very limited data available, and these show high deviations from the EOS.

**Table 1:** Review of Speed of Sound Data in Gaseous Helium Reported in the Literature Where *N* is the Number of Data Points, *T* is the Temperature, *p* is the Pressure, and Δ_AARD_ is the Absolute Average Relative Deviations of the Experimental Data Set from the Unpublished EOS (2015) of Ortiz Vega et al.^14^ as Calculated Using REFPROP 10^12^

Author(s)	Year	*N*	*T*/K	*p*/MPa	Δ_AARD_ (%)
Gammon^15^	1976	204	98–423	1–15	0.005
Hurly et al.^16^	1997	162	225–400	0.2–1.5	0.013
Van Itterbeek and Van Doninck^17^	1949	4	75–90	0.1	0.088
Van Itterbeek and Mariens^18^	1940	12	294–347	10^–5^–10–^4^	0.089
van Itterbeek and Thys^19^	1938	4	220–291	0.09–0.1	0.53
Kimura et al.^20^	1987	20	298	10–200	1.49
Pitaevskaya and Bilevich^21^	1970	168	298–473	40–400	2.48
Vidal et al.^22^	1980	10	298	100–1000	9.76
Hanayama and Kimura^23^	1995	20	295	52–3000	12.0
Kortbeek et al.^24^	1988	164	98–298	100–1000	12.3
Nishitake and Hanayama^25^	1975	15	298	137–1470	14.0
Curtis^26^	1934	2	273	0.1	14.6

The objective of the present work was to address the identified gap in the literature by providing new speed of sound data in gaseous helium at pressures from 15 to 100 MPa and at temperatures between 273 K and 373 K.

## 2 Experimental Section

The speed of sound measurements were carried out with the dual-path pulse-echo system described by Scholz et al.^27^ and Al Ghafri et al.^28^ In this method, an ultrasonic transducer is immersed in the fluid under study between two plane parallel end plates located at different distances. The transducer is excited to generate ultrasonic pulses that propagates through the fluid to each side. The pulses are reflected from the ends and return to the transducer, where the echoes are detected. The speed of sound is then determined by the time difference between the returning echoes and the difference between the lengths of the two paths.

The dual-path pulse-echo technique is a well-established technique for the determination of the speed of sound in liquids.^29−33^ However, it is generally considered that pulse techniques are not well suited to measurements on gases. This is because the acoustic impedance of the fluid (the product of the sound speed and the density) is generally low, leading to weak and possibly poorly defined ultrasonic pulses.^34^ However, Meier and Kabelac^35,36^ have shown that the dual-path pulse-echo technique can be applied successfully to compressed gases, leading to relative uncertainties in the measured speed of sound as small as 0.01% when the acoustic impedance is sufficient. Depending on the temperature, they reported clearly distinguishable ultrasonic signals at pressures ≥7 MPa for argon and ≥20 MPa for nitrogen. Dubberke et al.^37^ reported measurements at pressures as low as 5 MPa for argon and nitrogen.

**Figure 1:**
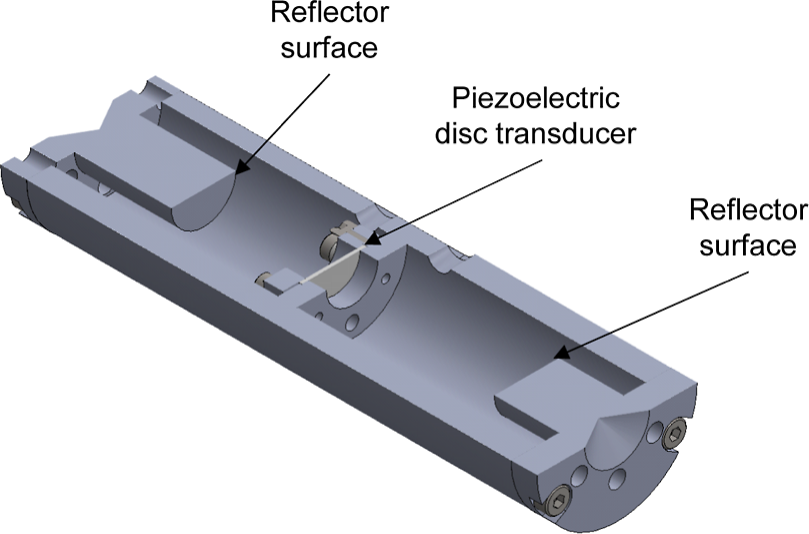
Cross-sectional view of the cylindrical ultrasonic cell.

As shown in Figure 1, the device used in this work consisted of a ceramic piezoelectric disc transducer (Piezo Technologies, type K360 with a diameter of 10 mm and a thickness of 0.4 mm) clamped perpendicular to the axis of a cylindrical ultrasonic cell. The cell was fabricated from Invar 36 nickel-iron alloy. Plane reflectors closed each end of the cell. According to the dual-path pulse-echo principle, the distances from the transducer disc to the two reflectors are different and, in the present work, the nominal lengths were *L*_1_ = 20 mm and *L*_2_ = 30 mm. To conduct a measurement, the transducer disc was excited by a three-cycle sinusoidal tone burst with a frequency of 5 MHz and an amplitude of 20 V_p-p_. This tone burst was generated by a function generator (Agilent, model 33220A), and the returning echoes were captured by a digital oscilloscope (Agilent, model DSO6012A). The ultrasonic cell was mounted in a pressure vessel rated for pressures up to 400 MPa. The pressure vessel was immersed in a temperature-controlled bath (Fluke, model 6020), which was filled with a water-ethylene glycol mixture. To achieve temperatures below 313 K, a copper heat exchanger, connected to a closed-circuit chiller (Huber, model CC-K6s), was immersed in the bath fluid. The pressure was regulated by using a manual syringe pump and measured by a pressure transducer (Keller, model 33X, 100 MPa full scale), both located in the piping outside of the bath. The temperature of the pressure vessel was measured with a platinum resistance thermometer (Fluke, model 5615) immersed in an axial thermometer well and connected to a resistance read-out unit (Fluke, model 1502A).

The speed of sound *c* was determined from the measured time delay Δ*t* between the echoes returning on the short and the long paths, for which the path difference is 2(*L*_2_ – *L*_1_), according to the equation
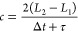
1Here, *τ* is a small correction for diffraction given by^38^
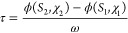
2where  is the diffraction phase advance, *S*_*i*_ = 2*L*_*i*_λ/*b*^2^, λ is the wavelength, *b* is the effective transducer radius, χ_*i*_ = 2*L*_*i*_/*b*, and ω is the angular frequency. In the present case, the clamping arrangement reduces the effective transducer radius to *b* = 4 mm.

The path length difference Δ*L* = (*L*_2_ – *L*_1_) is represented as a function of temperature and pressure as follows
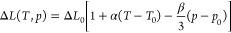
3Here, Δ*L*_0_ is the length difference at a reference temperature *T*_0_ and reference pressure *p*_0_, which was determined by a calibration measurement in gaseous nitrogen at *T*_0_ = 298.15 K and *p*_0_ = 70 MPa. Additionally, *α* is the mean thermal expansivity over the interval [*T*_0_, *T*], and *β* is the compressibility of the cell material. The thermal expansion coefficient of the Invar alloy was determined experimentally via dilatometry at the UK National Physical Laboratory at temperatures between 223 K and 473 K. From these results, the following correlation for the temperature-dependent mean thermal expansion coefficient was determined by Scholz et al.^27^
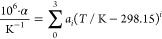
4

For the mean isothermal compressibility of Invar, Scholz et al.^27^ established a correlation based on isothermal bulk modulus values reported by Hausch and Warlimont^39^
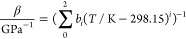
5

The parameters for both correlations are given in Table 2.

**Table 2:** Parameters of eq 4 for Mean Thermal Expansion Coefficients and of eq 5 for Mean Isothermal Compressibility^27^

	*i* = 0	*i* = 1	*i* = 2	*i* = 3
*a*_*i*_	1.650	8.552 × 10^–4^	2.941 × 10^–5^	4.397 × 10^–8^
*b*_*i*_	1.061 × 10^2^	3.155 × 10^–2^	3.722 × 10^–4^	

Before filling with gas, the vessel and connecting tubing were evacuated. Since the speed of sound in helium differs greatly from that in plausible impurities such as nitrogen and water vapor, impurities have a strong influence on measurements of the speed of sound in helium. Therefore, five filling-evacuation cycles of the vessel and the tubing were performed for both gases before the final gas samples were transferred into the system. For each cycle, the system was filled to a pressure of about 10 MPa with the gas under investigation and then exhausted to vacuum. The helium and nitrogen used are described in Table 3.

**Table 3:** Description of the Gas Samples Used for the Speed of Sound Experiments, Where *x* Denotes Mole Fraction

Gas	CAS number	Supplier	Purity *x*	Purification method
helium	7440-59-7	BOC	0.99999^a^	none
nitrogen	7727-37-9	BOC	0.999995^b^	none

a Impurities (stated by supplier): *x*(N_2_) ≤ 5.0 × 10^–6^, *x*(H_2_O) ≤ 2.0 × 10^–6^, *x*(O_2_) ≤ 2.0 × 10^–6^, *x*(H_2_) ≤ 1.0 × 10^–6^, *x*(CO_2_) ≤ 0.5 × 10^–6^, and *x*(C_*x*_H_*y*_) ≤ 0.5 × 10^–6^.

b Impurities (stated by supplier): *x*(H_2_O) ≤ 1.0 × 10^–6^, *x*(O_2_) ≤ 1.0 × 10^–6^, *x*(H_2_) ≤ 1.0 × 10^–6^, [*x*(CO) + *x*(CO_2_)] ≤ 1.0 × 10^–6^, and *x*(C_*x*_H_*y*_) ≤ 0.5 × 10^–6^.

The reference EOS of Span et al.^40^ was used to calculate the speed of sound in nitrogen. Span et al.^40^ state that the uncertainty in the speed of sound at our calibration conditions is 0.6%, which was a conservative estimation made in the absence of validation data. With this high estimated uncertainty, nitrogen would have not been a suitable calibration gas. However, Meier and Kabelac^36^ showed by measuring several isotherms from 275 to 400 K and pressures up to 100 MPa that the uncertainty of the EOS is actually much lower. For the isotherm measured at 300 K, close to our calibration temperature of 298.15 K, they report relative deviations from the EOS within ±0.01%. Due to this reason, the deviations reported by Meier and Kabelac^36^ from the nitrogen EOS of about ±0.01% were assumed as the relative standard uncertainty for the speed of sound *c*_0_ in nitrogen at calibration conditions.

The uncertainties of the measurements were estimated considering the working equation given by Scholz et al.,^27^ in which the path difference Δ*L* was eliminated in favor of the time difference Δ*t*_0_ and speed of sound *c*_0_ in nitrogen at calibration temperature *T*_0_ and pressure *p*_0_. The uncertainty contribution of the diffraction term was neglected, as the influence of *τ*, in general, is small. The standard uncertainty is given by
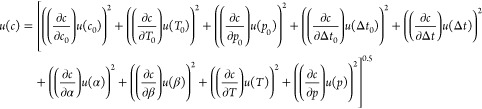
6

For the time differences, an amplitude-dependent standard uncertainty was considered, as it was observed that at low pressures (*i.e.,* small acoustic impedances), the weak echo signals influenced the repeatability of the measured time differences. To address this, the standard uncertainty of Δ*t* was related to the amplitude *A*_1_ of the first echo as follows
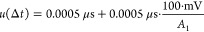
7

Due to different acoustic impedances, the amplitude measured depended on the gas under study and the pressure. In nitrogen, an amplitude of 127 mV was measured at a temperature of 298.15 K and a pressure of 30 MPa, while at the same temperature, an amplitude of 665 mV was measured at a pressure of 100 MPa. In helium, the smallest amplitude of 19 mV was measured at a temperature of 348.15 K and a pressure of 15 MPa, while the highest amplitude of 169 mV was measured at a temperature of 273.15 K and a pressure of 100 MPa. Therefore, the influence of the amplitude on the standard uncertainty of Δ*t* was significantly greater for helium than for nitrogen. For the thermal expansion coefficient and the isothermal compressibility, relative standard uncertainties of about 10 and 1%, respectively, were assumed as reported by Scholz et al.^27^ The standard uncertainty in the temperature measurement is 0.015 K.^27^ The standard uncertainty of pressure was estimated from the total uncertainty given by the manufacturer (0.05% of full scale) considered as a rectangular distribution. With a divisor of √3, this leads to a standard uncertainty in pressure of 0.03 MPa.

## 3 Results and Discussion

### 3.1 Calibration Measurements in Nitrogen

**Figure 2:**
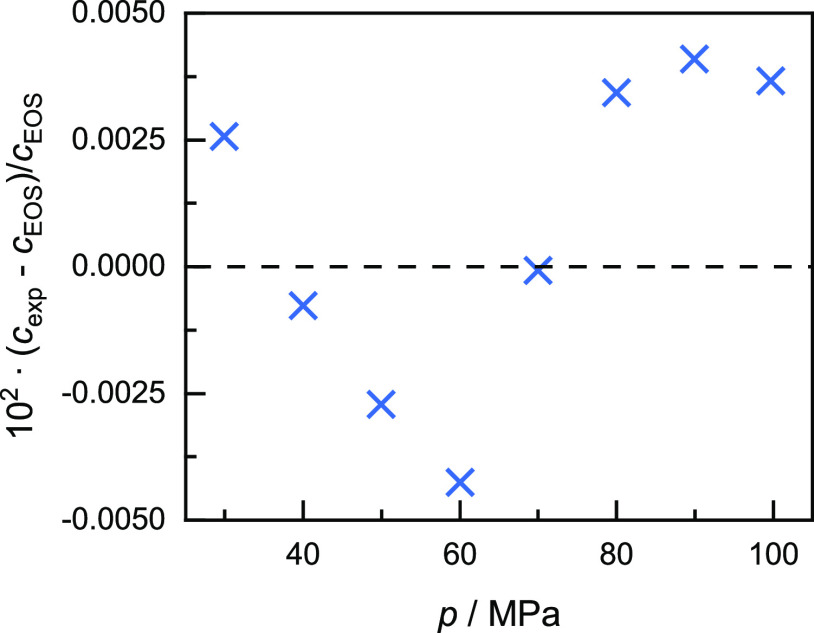
Relative deviations of the experimentally determined speed of sound values *c*_exp_ in nitrogen at a temperature of 298.15 K from the values calculated with the EOS (dashed line) of Span et al.^40^

As described above, a one-point calibration was performed with nitrogen gas at *T*_0_ = 298.15 K and *p*_0_ = 70 MPa. By adjusting Δ*L*_0_, the deviation of the measurement from the EOS of Span et al.^40^ was reduced to zero at that point, yielding 2Δ*L*_0_ = 19.6972 mm. Additional measurements were carried out along the same isotherm at pressures between 30 and 100 MPa. In all cases, a small correction was applied to account for the long vibrational relaxation time of nitrogen, as detailed by Meier and Kabelac.^36^ In Figure 2, relative deviations of the experimentally determined speed of sound values in nitrogen from the values calculated with the EOS of Span et al.^40^ are shown. Based on the calibration, the experimental speeds of sound are in excellent agreement with the EOS with all deviations within ±0.005%.

### 3.2 Measurements in Helium

**Figure 3:**
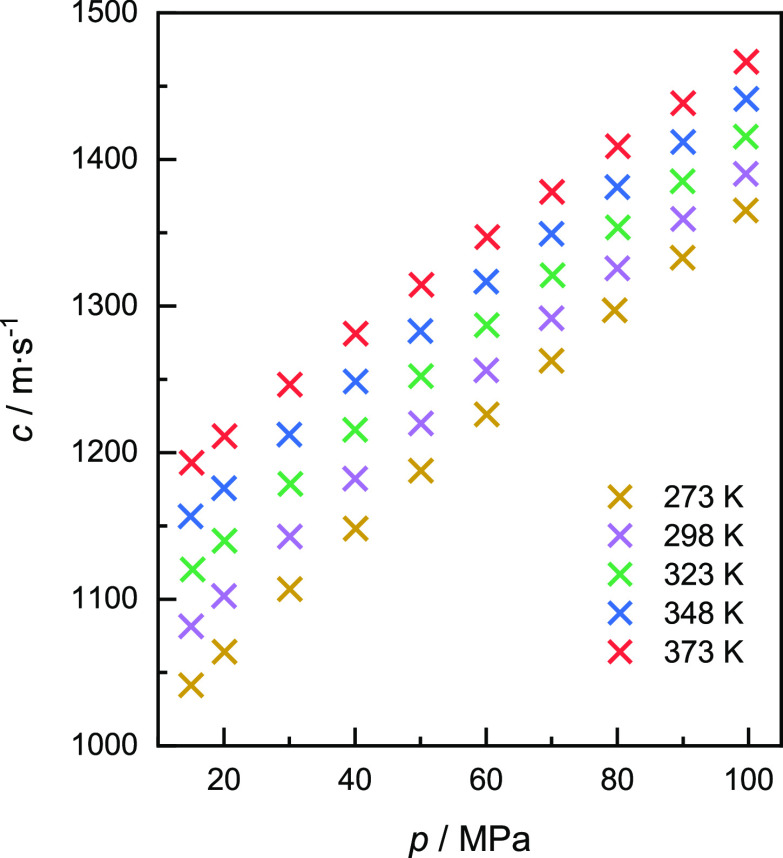
Measured speed of sound values in helium at five different temperatures.

The speed of sound in helium was measured along five isotherms at temperatures of (273, 298, 323, 348, and 373) K. Each isotherm consisted of ten different pressures, ranging from (15 to 100) MPa. Measurements below 15 MPa could not be analyzed properly, as the echoes were not clearly distinguishable. The experimental speeds of sound are shown as a function of pressure in Figure 3 and listed in Table 4 with the corresponding temperature *T*, pressure *p*, and expanded uncertainties *U*_c_(*c*). The speeds of sound increase with increasing temperature and increasing pressure consistently. The expanded uncertainties were based on eq 6 with a coverage factor *k* = 2. As an example, the uncertainty budget for the measurement at *T* = 323 K and *p* = 40 MPa is detailed in Table 5. Since, on the one hand, the uncertainty of the time differences is a function of the signal amplitude according to eq 7 and, on the other hand, the absolute speed of sound values is increasing with pressure, the values of the expanded uncertainty initially decrease and then increase with increasing pressure.

**Table 4:** Experimental Speed of Sound Data *c* in Helium with Expanded Uncertainties *U*(*c*) with a Coverage Factor of *k* = 2 at Temperature *T* and Pressure *p*^a^

*T*/K	*p*/MPa	*c*/(m·s^–1^)	*U*(*c*)/(m·s^–1^)	*T*/K	*p*/MPa	*c*/(m·s^–1^)	*U*(*c*)/(m·s^–1^)
273.157	14.998	1041.55	0.34	323.154	60.097	1287.27	0.34
273.160	20.117	1064.15	0.32	323.155	70.090	1321.24	0.35
273.160	30.100	1106.95	0.30	323.155	80.055	1353.90	0.36
273.160	40.111	1148.36	0.30	323.154	89.961	1385.49	0.36
273.160	50.040	1187.73	0.30	323.154	99.565	1415.62	0.35
273.162	60.063	1226.20	0.31	348.135	14.943	1156.64	0.49
273.160	69.949	1262.85	0.31	348.141	20.066	1175.85	0.42
273.161	79.566	1297.16	0.32	348.138	29.999	1212.61	0.34
273.161	89.988	1333.23	0.33	348.138	40.077	1248.63	0.33
273.160	99.569	1365.46	0.33	348.139	49.945	1283.11	0.34
298.153	15.049	1081.51	0.39	348.139	60.008	1316.73	0.34
298.153	20.053	1102.39	0.35	348.137	69.999	1349.35	0.34
298.154	30.061	1143.06	0.33	348.137	80.000	1381.46	0.34
298.152	40.046	1182.23	0.33	348.140	90.039	1412.15	0.35
298.151	50.016	1219.97	0.41	348.142	99.657	1441.39	0.36
298.153	59.989	1256.45	0.33	373.147	15.061	1193.40	0.44
298.154	69.979	1291.79	0.34	373.148	20.080	1211.48	0.43
298.152	79.978	1326.03	0.34	373.148	30.068	1246.54	0.37
298.154	90.017	1359.44	0.34	373.151	40.133	1281.26	0.35
298.153	99.567	1390.42	0.35	373.150	50.151	1314.55	0.35
323.153	15.234	1120.50	0.38	373.150	60.131	1347.24	0.35
323.157	20.108	1139.91	0.35	373.153	70.004	1377.99	0.35
323.155	30.113	1178.65	0.33	373.150	80.113	1409.27	0.36
323.154	40.056	1215.84	0.32	373.153	89.994	1438.45	0.36
323.153	50.060	1252.14	0.35	373.146	99.653	1466.72	0.37

a Standard uncertainties are *u*(*T*) = 0.015 K and *u*(*p*) = 0.03 MPa.

**Table 5:** Uncertainty Budget for the Speed of Sound in Helium at a Pressure of *p* = 40.056 MPa and a Temperature of *T* = 323.154 K, in Which *c*_0,EOS_ is the Calculated Speed of Sound in Nitrogen at Calibration Conditions, Δ*t*_0_ is the Time Difference at Calibration Conditions, Δ*t* is the Time Difference at Measurement Conditions, *α* is the Mean Thermal Expansion Coefficient over the Interval [*T*_0_, *T*], and *β* is the Isothermal Compressibility at Measurement Conditions^a^

Quantity	Value	Standard uncertainty	Sensitivity coefficient	Uncertainty contribution
*c*_0,EOS_	707.34 m·s^–1^	0.0707 m·s^–1^	1.719	0.122 m·s^–1^
Δ*t*_0_	27.8421 μs	0.0005 μs	43.664 m·s^–1^·μs^–1^	0.022 m·s^–1^
*T*_0_	298.152 K	0.015 K	0.069 m·s^–1^·K^–1^	0.001 m·s^–1^
*p*_0_	69.961 MPa	0.03 MPa	1.43 m·s^–1^·MPa^–1^	0.041 m·s^–1^
Δ*t*	16.2016 μs	0.0012 μs	75.036 m·s^–1^·μs^–1^	0.091 m·s^–1^
*α*	1.65 × 10^–6^ K^–1^	1.65 × 10^–7^ K^–1^	3.04 × 10^4^ m·s^–1^·K	0.005 m·s^–1^
*β*	9.33 × 10^–6^ MPa^–1^	9.33 × 10^–8^ MPa^–1^	1.2 × 10^4^ m·s^–1^·MPa	0.001 m·s^–1^
*T*	323.154 K	0.015 K	0.199 m·s^–1^·K^–1^	0.003 m·s^–1^
*P*	40.056 MPa	0.03 MPa	1.06 m·s^–1^·MPa^–1^	0.031 m·s^–1^

a Combined expanded uncertainty (*k* = 2) of *c* = 0.32 m·s^–1^.

### 3.3 Comparison of Measurements with the Helmholtz Equation of State

**Figure 4:**
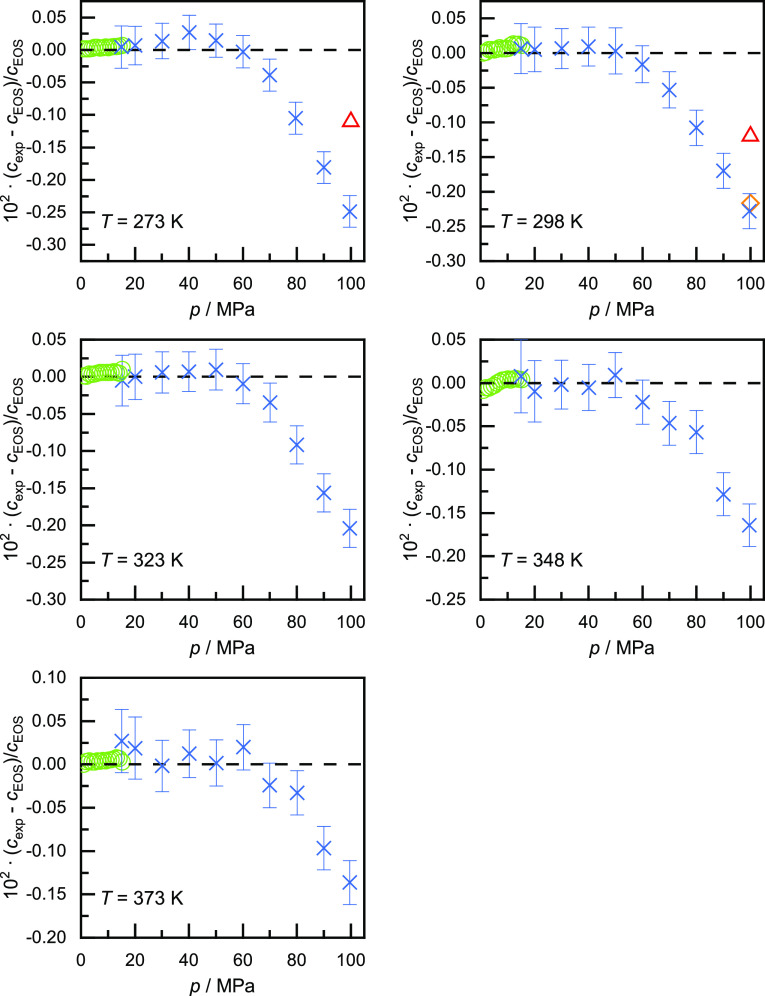
Relative deviations of the experimentally determined speeds of sound *c*_exp_ in helium at five different temperatures from the values calculated with the unpublished EOS of Ortiz Vega et al. (dashed line):^14^ ×, this work; ◯, Gammon;^15^◇, Vidal et al.,^22^ △, Kortbeek et al.^24^

In Figure 4, we compare the experimental speeds of sound with the unpublished EOS of Ortiz Vega et al.^14^ as implemented in REFPROP 10 along each isotherm individually. The experimental values are shown with the corresponding relative expanded uncertainty. In addition, we also plot the comprehensive data set reported by Gammon,^15^ two data points reported by Kortbeek et al.,^24^ and one data point reported by Vidal et al.^22^ For the five investigated isotherms, the deviations from the EOS show a consistent trend. Up to pressures of 50 to 60 MPa, the experimental values are in very good agreement with the EOS, with deviations within ±0.03%, which is within the expanded uncertainty of the measurements. With further pressure increases, negative deviations of increasing magnitude can be observed. The worst deviations, observed at the greatest pressure, improve progressively with increasing temperature from −0.26% at *T* = 273 K to −0.15% at *T* = 373 K.

The data of Gammon,^15^ who measured up to 15 MPa, agree very well with the EOS. This is no surprise as this is one of the two primary data sets used for the development of the EOS. But as the data are also in very good agreement with the present results, they can be interpreted as validating the presented work at *p* = 15 MPa. Vidal et al.^22^ reported one data point at *p* = 100 MPa and *T* = 298 K, which is also in very good agreement with the present work. Measurements at higher pressure by Vidal et al.^22^ also follow this consistent trend (not shown here). The two data points reported by Kortbeek et al.^24^ at *p* = 100 MPa and *T* = (273 and 298) K, respectively, show also negative deviations from the EOS, but at around -0.11%, their values are closer to the calculated values than ours. However, Kortbeek et al.^24^ report that the lowest pressure at which they could measure was 130 MPa, and the values reported at 100 MPa were obtained by extrapolation. Hanayama and Kimura,^23^ Kimura et al.,^20^ and Pitaevskaya and Bilevich^21^ reported data in the pressure range up to 100 MPa as well, but the deviations of their data from the EOS range in that pressure regime are between -2.1 and +5.5% and show no clear pressure-depending trend, which is the reason why they are not shown in Figure 4. These large deviations might also be a reason why the data sets were not considered for the development of the EOS.

### 3.4 Comparison with Data from *Ab Initio* Acoustic Virial Coefficients

The EOS of helium is amenable to theoretical evaluation based on *ab initio* interatomic potential-energy functions. Starting from such interatomic potentials, one can evaluate the temperature-dependent virial coefficients *B*_*n*_ in the virial EOS (VEOS)
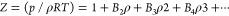
8Here, *Z* is the compressibility factor, *ρ* is the molar density, and *B*_*n*_ is the *n*^th^ temperature-dependent virial coefficient. The second virial coefficient *B*_2_ rigorously depends only upon the interatomic pair-potential-energy function, while the higher-order virial coefficients involve many-body interactions and require non-additive corrections to the interatomic potential energy. The virial EOS leads in turn to a similar power-series expansion for the “acoustic compressibility factor” *Z*_a_ as follows
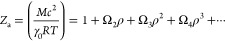
9Here, *γ*_0_ = 5/3, and Ω_*n*_ is the *n*^th^ acoustic virial coefficient, which is related to the corresponding, and all lower order, ordinary virial coefficients *B*_*n*_ and their first two temperature derivatives. The EOS in this form has been studied in detail by Gokul et al.^41^ on the basis of the *ab initio* pair potential of Przybytek et al.^42,43^ and the non-additive three-body correction of Cencek et al.^44^ Gokul et al.^41^ present precise calculations of the acoustic virial coefficient up to order *n* = 7, estimates of the statistical uncertainty of each coefficient, and correlations of both *B*_*n*_ and Ω_*n*_ as functions of temperature.

**Figure 5:**
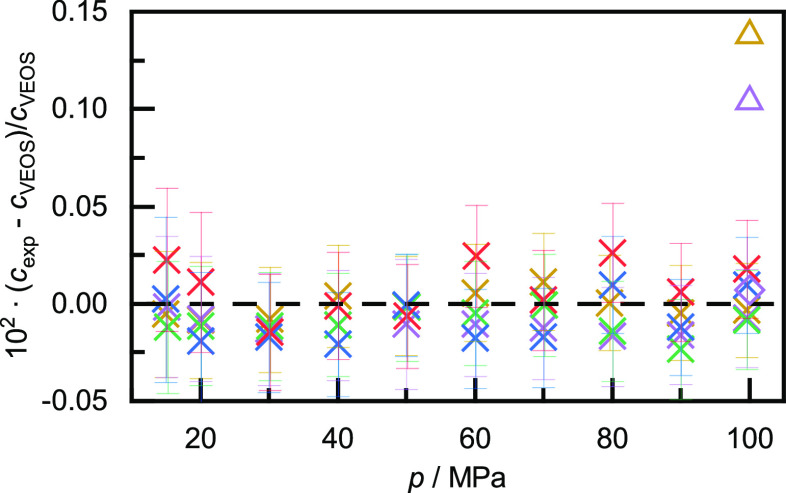
Relative deviations of the experimentally determined speeds of sound *c*_exp_ in helium from the values calculated with the *ab initio* VEOS by Gokul et al. (dashed line).^41^ ×, this work; ◇, Vidal et al.,^22^ △, Kortbeek et al.^24^ Colors denote temperature: red = 373 K; blue = 348 K; green = 323 K; purple = 298 K; brown = 273 K.

To apply this EOS, we first solved eq 8 for the molar density corresponding to the experimental temperatures and pressure and then used eq 9 to evaluate the speed of sound using, in both equations, coefficients up to *n* = 7 as correlated by Gokul et al.^41^ We did not make use of the pressure-series expansion of *Z*_a_ reported by Gokul et al.^41^ because it does not appear to converge at the highest pressures studied in this work. On the other hand, both eqs 8 and 9 appear to converge to this order to within 0.01%, as judged by the difference between truncation after *n* = 6 and after *n* = 7. Furthermore, the uncertainties of the virial coefficients as reported by Gokul et al.^41^ give rise to uncertainties in *Z* or *Z*_a_ which are smaller than 0.015%. Figure 5 compares our experimental speeds of sound with those calculated from the virial EOS and, remarkably, the agreement is within the experimental uncertainty over the entire pressure range. Also plotted in Figure 5 are the values at *p* = 100 MPa reported by Vidal et al.^22^ and by Kortbeek et al.;^24^ the former also showing excellent agreement with the acoustic virial EOS.

## 4 Conclusions

The speed of sound in gaseous helium was measured along five isotherms from (273 to 373) K and in a pressure range from (15 to 100) MPa using the dual-path pulse-echo technique. This new data set fills a significant gap in the database of the thermodynamic properties of helium. The expanded uncertainty of the speed of sound measurements ranges from (0.30 to 0.49) m·s^–1^, fractionally (0.02 to 0.04)%. At pressures up to 50 MPa, the data agree within the experimental uncertainty with the *de facto* reference EOS, the unpublished equation of Ortiz Vega et al.,^14^ implemented in common thermophysical property software such as REFPROP 10 and TREND 5.0. At higher pressures, negative deviations of increasing magnitude are consistently observed, with the worst case being a relative deviation of -0.26% at *T* = 273 K and *p* = 100 MPa. Since Ortiz Vega et al.^14^ considered data only up to 15 MPa, the EOS was not previously validated for higher pressures. It can now be concluded that the uncertainty of the Ortiz Vega et al. EOS at pressures above 60 MPa is significantly greater than the estimates of 0.01% given in REFPROP 10 and TREND 5.0. The new experimental data may be an important contribution in the future to the optimization of a new wide-ranging EOS for helium. Remarkably, the present results agree with the predictions of the virial EOS, with the ordinary and acoustic virial coefficients reported by Gokul et al.,^41^ at all investigated state points.
